# A real-world assessment of healthcare resource utilization following IBS-Smart ^®^ and Trio-Smart ^®^ testing in patients with suspected irritable bowel syndrome

**DOI:** 10.3389/fgstr.2026.1681818

**Published:** 2026-07-23

**Authors:** Leonard Weinstock, William B. Salt, Lynn Cherry, Christopher Tyson, Raf Magar

**Affiliations:** 1 Gastrointestinal Alliance, St. Louis, MO, United States; 2 IBS and Gut Microbiome Solutions, Dublin, OH, United States; 3 AHRM Inc., Buffalo, NY, United States; 4 AHRM Inc., Raleigh, NC, United States

**Keywords:** rain interaction, IBS-Smart, Trio-Smart, healthcare utilization, cost, breath testing

## Abstract

**Background:**

IBS is a disorder of gut-brain interaction that is diagnosed positively but still requires a focused differential diagnosis and limited rule-out testing. IBS-Smart and Trio-Smart are commercially available serologic and breath tests that may alter diagnostic sequencing in clinical practice.

**Objective:**

To evaluate whether receipt of IBS-Smart and/or Trio-Smart was associated with differences in diagnostic resource utilization, captured diagnostic cost, chart-level diagnostic persistence, and medication use compared with contemporaneous control patients who did not receive these tests.

**Methods:**

This retrospective two-center cohort study included 219 adults with symptoms suggestive of IBS. Patients were grouped as IBS-Smart only (n=48), Trio-Smart only (n=43), both tests (n=11), or controls receiving neither test (n=117). Costs reflected a payer-proxy perspective in 2022 US dollars and included captured diagnostic tests and gastroenterology office visits. The archived study used propensity matching and an exploratory generalized linear model (GLM) for total diagnostic cost.

**Results:**

Colonoscopy rates were 50.0% in the IBS-Smart-only cohort, 53.5% in the Trio-Smart-only cohort, 27.3% in the combined-testing cohort, and 60.7% in controls; upper endoscopy rates were 31.3%, 41.9%, 0.0%, and 48.7%, respectively. Among patients first seen in 2018 or later, mean total captured diagnostic plus office-visit cost per patient per month was $366.85 for IBS-Smart only, $382.87 for Trio-Smart only, $361.83 for both tests, and $960.39 for controls. In the archived propensity-matched GLM, IBS-Smart testing was associated with lower total diagnostic cost (coefficient -0.22; P = 0.050), corresponding to an estimated incremental savings of $526 per patient. Stable chart-level diagnosis was observed in 79.2% of IBS-Smart-only patients, 90.7% of Trio-Smart-only patients, 72.7% of patients after IBS-Smart in the combined cohort, 90.9% after Trio-Smart in the combined cohort, and 25.6% of controls. Medication counts decreased descriptively in several tested subgroups at one year; drug-class-level aggregation could not be reconstructed reliably from the archived medication file and is therefore not overstated.

**Conclusions:**

In this observational study, receipt of IBS-Smart and/or Trio-Smart was associated with lower captured resource utilization and greater chart-level diagnostic persistence than standard care alone. These findings should be interpreted as real-world associations rather than causal proof and do not replace Rome IV-based evaluation, alarm-feature triage, or guideline-recommended rule-out testing.

## Introduction

Irritable bowel syndrome (IBS) is a common disorder of gut-brain interaction (DGBI) characterized by recurrent abdominal pain associated with defecation and/or a change in stool frequency or form (1, 2). In the United States, Rome IV IBS affects roughly 6% of adults and is associated with substantial symptom burden, healthcare utilization, and reduced quality of life (3, 4). Contemporary IBS care therefore emphasizes a positive clinical diagnosis supported by focused investigations considering differential diagnosis of disorders and diseases with the IBS phenotype, rather than an open-ended diagnosis of exclusion (1–3).

At the same time, IBS is clinically heterogeneous. Visceral hypersensitivity altered central pain modulation, stress-related symptom amplification, motility disturbances, immune activation, bile acid abnormalities, pelvic floor dysfunction, and microbial perturbations may all contribute to symptoms in different patients (3, 5, 6). This biopsychosocial and gut-brain framework is important because no single biomarker captures the full syndrome, and a positive diagnosis does not eliminate the need to consider alarm features and a limited differential diagnosis when diarrhea, weight loss, bleeding, inflammatory symptoms, or other red flags are present (2, 3, 7, 8).

For patients with suspected IBS and diarrhea symptoms, current guidelines recommend a targeted rule-out strategy that commonly includes celiac serology and fecal inflammatory markers such as fecal calprotectin or C-reactive protein (CRP), with colonoscopy, stool studies, or other testing reserved for selected patients based on alarm features or the clinical scenario (2, 7, 8). Chronic diarrhea can also reflect inflammatory bowel disease (IBD), microscopic colitis, bile acid diarrhea, pancreatic disease, medication effects, or selected infections and parasites. Blastocystis and other enteric pathogens may mimic IBS-like symptoms in some settings, although the clinical importance varies by context (8, 9).

Microbial and post-infectious mechanisms have also received growing attention in IBS. Anti-cytolethal distending toxin B (anti-CdtB) and anti-vinculin antibodies were developed to help differentiate diarrhea-predominant IBS (IBS-D) from IBD in patients with chronic diarrhea, whereas breath testing is used in selected patients to evaluate hypothesis-driven contributors such as small intestinal bacterial overgrowth (SIBO) or intestinal methanogen overgrowth (IMO) (10–13). However, terms such as dysbiosis and intestinal sulfide overproduction (ISO) should be interpreted cautiously. Dysbiosis has no universally accepted clinical definition, SIFO was not assessed in this study, and ISO remains an emerging breath-test phenotype rather than a universally accepted end-diagnosis (6, 12–15).

IBS-Smart is a commercially available blood test that incorporates anti-CdtB and anti-vinculin assays, and Trio-Smart is a three-gas breath test that reports hydrogen, methane, and hydrogen sulfide. Both tests are less invasive than colonoscopy or upper endoscopy and may alter diagnostic sequencing in practice. The question addressed by the present study was not whether these tests replace clinical reasoning, but whether their use in real-world specialty care was associated with differences in diagnostic resource utilization, chart-level diagnostic persistence, and medication use when compared with contemporaneous patients who did not receive these tests.

## Methods

### Study design

**Figure 1:**
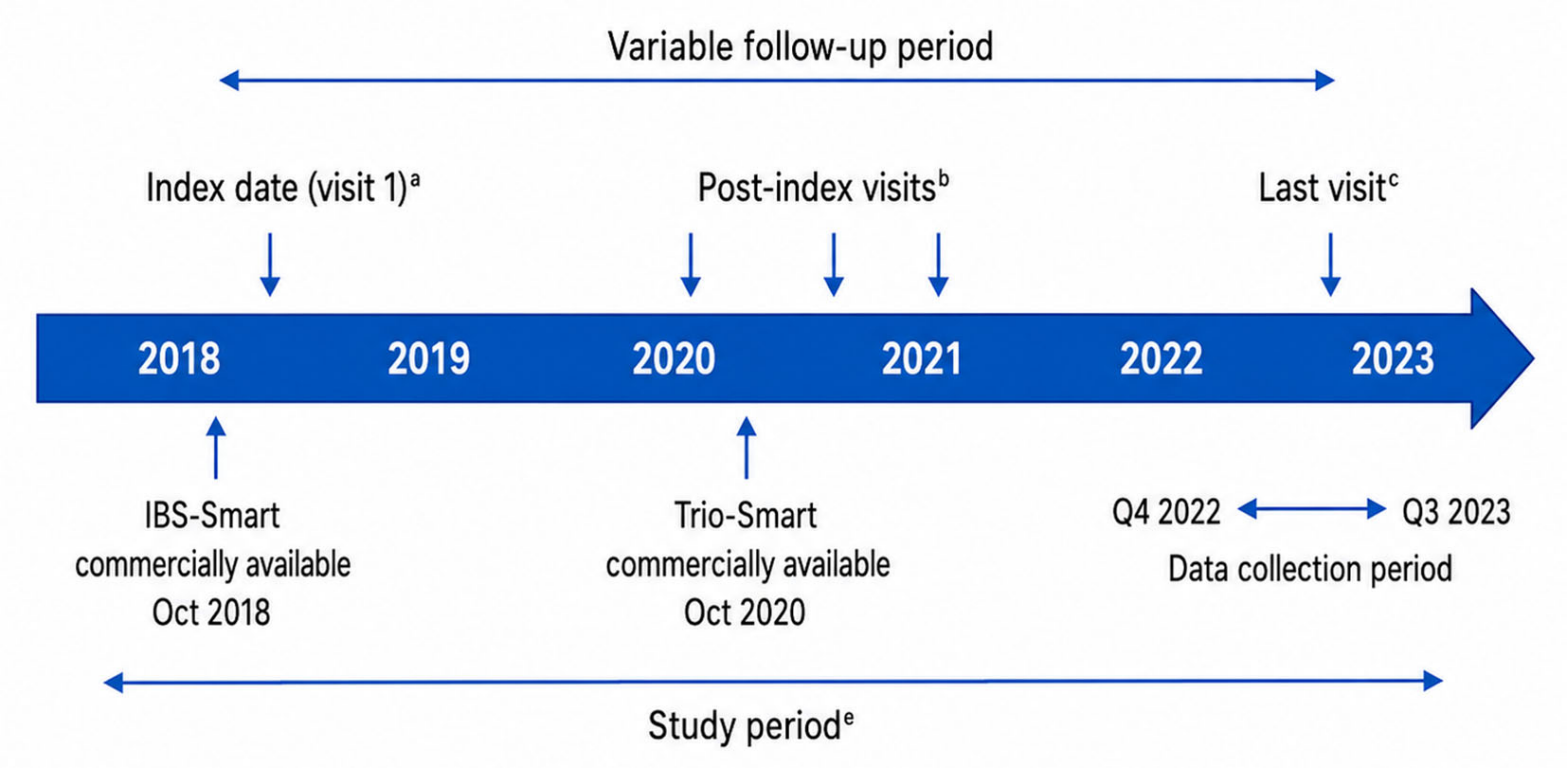
Study design. **(a)**. Index is the patient’s first office visit, recorded by month and year of visit; **(b)**. Postindex visits were recorded according to days from index date; patients had a minimum of 3 visits (including the index and last visits); **(c)**. Last visit during the study period denotes end of the variable follow-up period; **(d)**. For each patient, medical records were reviewed from their first to last visit within the study period; **(e)**. Study period was defined by the year of the earliest patient visit to the last patient visit reviewed in the study; specific dates were not recorded to maintain patient confidentiality.

For this retrospective cohort study, medical records were reviewed for patients suspected of IBS-D/M who had visited either of two centers in the United States, Specialists in Gastroenterology (St. Louis, MO) or IBS and Gut Microbiome Solutions (Dublin, OH). Records were eligible for inclusion beginning in 2018, when the IBS-Smart test became commercially available, through the start of the data collection period (August 2022 to November 2023, **Figure 1**). For each patient, the first visit to the study site with symptoms suggestive of IBS-D/M is the Index date. Patient history dated back as far as 2013. All data were collected using an electronic case report form (eCRF), which was completed by study site personnel and de-identified.

The protocol was reviewed and approved by the Salus Institutional Review Board. Informed consent was not obtained as exemption by full waiver was requested and in accordance with the US Department of Health and Human Services “Safe Harbor” method.

### Patient population

Adults aged 18 years or older were eligible if they had symptoms suggestive of IBS, including abdominal pain, stomach cramping, gas, bloating, chronic diarrhea, alternating bowel habits, and/or constipation; had at least three visits and at least three months of follow-up; and had a documented gastrointestinal diagnosis in the reviewed chart. Patients with pregnancy or alarm symptoms such as fever, hematochezia, or hematemesis were excluded. Because this was an observational chart review, no prospective diagnostic protocol was mandated before labeling a patient as having symptoms suggestive of IBS. Instead, rule-out tests were captured in the charts and interprets the findings within that limitation.

Patients were categorized into four described cohorts for descriptive analysis: IBS-Smart only, Trio-Smart only, both IBS-Smart and Trio-Smart, and a contemporaneous control group that received neither test. For the control group, additional charts meeting the same inclusion and exclusion criteria were reviewed in randomly generated order.

### Outcomes

The primary economic outcome was total diagnostic cost, defined as the sum of captured diagnostic test costs and gastroenterology office-visit costs. The costing perspective was a payer-proxy perspective based on allowed amounts rather than true societal cost, and the price year was 2022 US dollars. For most diagnostic tests, costs were assigned using Current Procedural Terminology-based amounts from the New Hampshire Comprehensive Health Care Information System database (16). When a specific test was not available in that database, public cash-price sources or manufacturer-listed prices were used. Both IBS-Smart and Trio-Smart costs were included.

### Data collection

Data were abstracted into an electronic case report form capturing demographics, smoking history, weight, comorbidities, referral status, presenting symptoms, year of first visit, diagnostic tests and procedures, diagnoses recorded throughout follow-up, and medications with available start and stop dates. Diagnostic and utilization variables were retained exactly as abstracted from the original chart. A limitation of the chart-based approach is that diagnoses relied in part on chart coding and clinician documentation, which may be influenced by reimbursement practices and local coding habits.

### Statistical analyses

Descriptive statistics were reported for demographic and clinical characteristics; diagnostic test use, office visits, and associated costs; diagnostic stability; and medication use. Age was calculated at the first visit (Index date). For patients with year of first visit 2018 or later (based on approval date of IBS-Smart), tests per patient per month, office visits per patient per month, and their respective costs per patient per month, were calculated based on the number of days each individual patient was followed.

Direct group-versus-control comparisons for colonoscopy and upper endoscopy rates, absolute risk differences, and the approximate number needed to test (NNT) to avert one procedure. These NNT values are descriptive rather than causal because treatment selection was not randomized. For analysis of medication use, identification of “brand” medications was based on whether goodrx.com (17) classified a medication as brand or generic.

Chart-level diagnostic persistence was defined as the last diagnosis before a test was compared with the first diagnosis after the test. If a diagnosis was present before the test but not after, the chart was counted as stable. For control patients, an analogous change in diagnosis could occur anywhere in the available chart history because no test date existed. This metric should therefore be interpreted as diagnostic persistence in documentation rather than as diagnostic accuracy.

A generalized linear model (GLM) was used to examine the effect of IBS-Smart testing on total diagnostic costs. For the GLM analysis, nearest neighbor propensity matching was performed on key baseline variables including age, whether patients had mast cell activation syndrome (MCAS) or IBS-D on their first visit, whether patients were referred, and whether their first visit was before 2019 (since the IBS-Smart test became available in late 2018), to match the demographics of the control and intervention groups in approximately a 1:1 ratio.

The dependent outcome variable in the GLM model was the total cost of diagnosis (testing and office visit costs). Predictor variables consisted of IBS-Smart testing and demographic or symptom variables that are clinically meaningful or show significant variance. Descriptive statistics and t tests were used to identify independent variables representing significant variance between cohorts and related to a diagnosis of IBS. Missing data were not imputed. Sample size calculations for a GLM estimated that a minimum of 120 patients would detect effect sizes (treatment population squared correlation coefficients) of at least 0.147 and 0.120 at power of 0.90 and 0.80, respectively. All analyses were performed using the R statistical programming environment.

## Results

### Patient population

**Figure 2:**
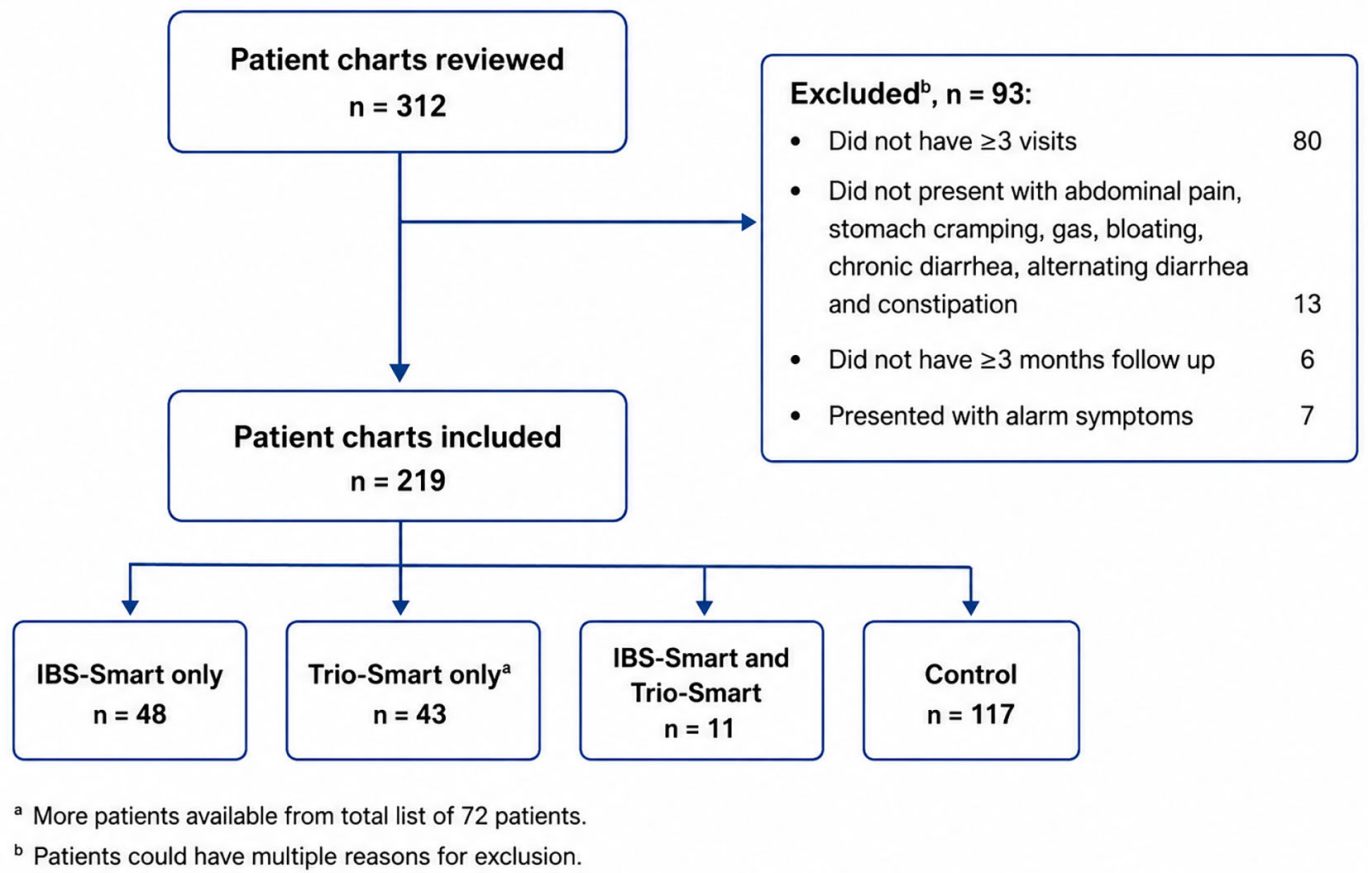
Patient flow chart. **(a)**. More patients available from list of 72 patients. **(b)**. Patients could have multiple reasons for exclusion.

A total of 312 patient charts were reviewed, of which, 219 patients met the study criteria and were included in the analysis (**Figure 2**). A subset of 48 patients received IBS-Smart only, 43 received Trio-Smart only, and 11 received both IBS-Smart and Trio-Smart; 117 patients receiving neither test were included in the control group. Patients were followed for a mean (SD) of 2.9 (1.8) years from Index date to last visit with the gastroenterologist. Demographic characteristics were broadly similar across cohorts, although control patients were somewhat older on average and were more frequently first seen in 2018 or earlier, reflecting earlier chart availability.

Presenting symptoms were consistent with an IBS-like population. Across cohorts, abdominal cramping, bloating, constipation, and diarrhea were common. Anxiety, depression, fatigue, and migraine were also frequent comorbidities. (**Table 1** and **2**). The clinical rule-out framework that would typically lead to a positive IBS diagnosis in current practice is shown in **Table 3**.

**Table 1:** Demographic characteristics by testing cohort (new).

Characteristic	IBS-Smart only (n=48)	Trio-Smart only (n=43)	Both tests (n=11)	Control (n=117)
Age, mean (SD)	48.6 (16.17)	51.4 (17.47)	48.3 (13.57)	53.1 (19.06)
Years followed, mean (SD)	2.5 (1.54)	3.2 (1.87)	2.1 (1.16)	3.2 (1.85)
Female sex, n (%)	35 (72.9)	32 (74.4)	10 (90.9)	95 (81.2)
Asian, n (%)	3 (6.3)	0	1 (9.1)	2 (1.7)
Black or African American, n (%)	0	2 (4.7)	1 (9.1)	2 (1.7)
White, n (%)	45 (93.8)	41 (95.3)	9 (81.8)	113 (96.6)
Hispanic or Latino, n (%)	0	0	0	3 (2.6)
Not Hispanic or Latino, n (%)	47 (97.9)	43 (100.0)	11 (100.0)	110 (94.0)
Ethnicity not available, n (%)	1 (2.1)	0	0	4 (3.4)

**Table 2:** Clinical characteristics and presenting symptoms by testing cohort (new).

Characteristic	IBS-Smart only (n=48)	Trio-Smart only (n=43)	Both tests (n=11)	Control (n=117)
Weight, kg, mean (SD)	151.9 (41.39)	156.4 (35.73)	135.5 (21.78)	159.2 (42.84)
Never smoked, n (%)	39 (81.3)	38 (88.4)	10 (90.9)	92 (78.6)
Former smoker, n (%)	6 (12.5)	5 (11.6)	0	15 (12.8)
Current smoker, n (%)	3 (7.0)	0	1 (9.1)	10 (8.5)
Anxiety, n (%)	23 (47.9)	19 (44.2)	2 (18.2)	42 (35.9)
Bulimia, n (%)	0	0	0	1 (0.9)
Depression, n (%)	13 (27.1)	9 (20.9)	1 (9.1)	29 (24.8)
Diabetes, n (%)	1 (2.1)	1 (2.3)	0	8 (6.8)
Fatigue, n (%)	21 (43.8)	19 (44.2)	3 (27.3)	26 (22.2)
Functional dyspepsia, n (%)	0	0	0	1 (0.9)
Fibromyalgia, n (%)	3 (6.3)	1 (2.3)	0	10 (8.5)
Migraine, n (%)	9 (18.8)	16 (37.2)	4 (36.4)	33 (28.2)
Year of first visit 2018 or earlier, n (%)	18 (37.5)	18 (41.9)	2 (18.2)	85 (72.6)
2019, n (%)	6 (12.5)	7 (16.3)	3 (27.3)	15 (12.8)
2020, n (%)	11 (22.9)	7 (16.3)	4 (36.4)	9 (7.7)
2021, n (%)	11 (22.9)	6 (14.0)	1 (9.1)	7 (6.0)
2022, n (%)	2 (4.2)	5 (11.6)	1 (9.1)	1 (0.9)
Abdominal cramping at first visit, n (%)	34 (70.8)	36 (83.7)	7 (63.6)	91 (77.8)
Belching, n (%)	8 (16.7)	7 (16.3)	3 (27.3)	16 (13.7)
Bloating, n (%)	35 (72.9)	36 (83.7)	11 (100.0)	77 (65.8)
Chest pain, n (%)	2 (4.2)	5 (11.6)	0	7 (6.0)
Constipation, n (%)	28 (58.3)	22 (51.2)	5 (45.5)	68 (58.1)
Diarrhea, n (%)	38 (79.2)	33 (76.7)	6 (54.5)	75 (64.1)
Flatulence, n (%)	21 (43.8)	19 (44.2)	8 (72.7)	46 (39.3)
Halitosis, n (%)	5 (10.4)	2 (4.7)	2 (18.2)	5 (4.3)
Heartburn, n (%)	14 (29.2)	10 (23.3)	2 (18.2)	29 (24.8)
Nausea and vomiting, n (%)	17 (35.4)	17 (39.5)	2 (18.2)	42 (35.9)
Rectal pain or bleeding, n (%)	1 (2.1)	0	0	0
MCAS at first visit, n (%)	0	2 (4.7)	0	9 (7.7)
IBS-D at first visit, n (%)	2 (4.2)	1 (2.3)	0	9 (7.7)
Referred, n (%)	35 (72.9)	33 (76.7)	8 (72.7)	103 (88.0)

**Table 3:** Differential-diagnosis and minimum rule-out framework relevant to the present study (new).

Clinical consideration	Guideline-based relevance	Availability in archived chart review
Alarm features	Weight loss, overt GI bleeding, anemia, fever, nocturnal symptoms, family history or other red flags	Alarm symptoms were exclusion criteria, but individual red-flag elements were not uniformly abstracted beyond the exclusion screen.
Celiac disease	Serologic testing is recommended in IBS with diarrhea symptoms (2, 8)	Captured as a celiac panel and quantified in **Table 4**.
Inflammatory bowel disease	Fecal calprotectin and/or CRP recommended when diarrhea is present and alarm features are absent (2, 8)	CRP and fecal calprotectin were captured; colonoscopy was also captured. Histology-level adjudication was not standardized.
Microscopic colitis	Consider in chronic watery diarrhea, often via colonoscopy with biopsies	Colonoscopy was captured, but biopsy indication and histopathology were not uniformly abstracted.
Bile acid diarrhea	Consider when diarrhea persists or is treatment-refractory (3, 7)	Not prospectively standardized in the archived abstraction.
Infectious or parasitic diarrhea	Stool studies when clinically indicated; parasites such as Blastocystis may mimic IBS-like symptoms in some settings (9)	Not uniformly abstracted in the archived dataset.
SIBO/IMO/ISO or carbohydrate-related symptoms	Hypothesis-driven breath testing in selected patients (12, 13)	Lactulose breath testing and Trio-Smart were captured; SIFO was not assessed.

**Table 3** was added in revision to address reviewer requests for a clearer differential framework. It clarifies what the archived study could and could not quantify uniformly for every patient.

### Diagnostic resource utilization and costs

Abstracted data (**Table 4**) reveal celiac serology was recorded in 43.8% of the IBS-Smart-only cohort, 18.6% of the Trio-Smart-only cohort, 27.3% of the combined-testing cohort, and 12.0% of controls. CRP was recorded in 18.8%, 9.3%, 9.1%, and 7.7%, respectively, while fecal calprotectin was recorded in 8.3%, 2.3%, 9.1%, and 0.9%. Colonoscopy and upper endoscopy were also frequently used, emphasizing that these proprietary tests did not replace standard GI evaluation but instead appeared to coexist with a broader diagnostic work-up.

**Table 4:** Diagnostic tests received and assigned unit costs (new).

Test or procedure	Unit cost, USD	IBS-Smart only	Trio-Smart only	Both tests	Control
Capsule endoscopy	2711	1 (2.1)	5 (11.6)	0	4 (3.4)
CT scan	2097	0	0	0	2 (1.7)
Colonoscopy	1844	24 (50.0)	23 (53.5)	3 (27.3)	71 (60.7)
Upper endoscopy	1781	15 (31.3)	18 (41.9)	0	57 (48.7)
Trio-Smart	319	0	43 (100.0)	11 (100.0)	0
IBS-Smart	220	48 (100.0)	0	11 (100.0)	0
Sucrose breath test	210	4 (8.3)	3 (7.0)	2 (18.2)	1 (0.9)
IBSchek	199	1 (2.1)	1 (2.3)	0	4 (3.4)
Lactulose breath test	199	40 (83.3)	26 (60.5)	5 (45.5)	86 (73.5)
Celiac panel	180	21 (43.8)	8 (18.6)	3 (27.3)	14 (12.0)
Fecal calprotectin	91	4 (8.3)	1 (2.3)	1 (9.1)	1 (0.9)
C-reactive protein	84	9 (18.8)	4 (9.3)	1 (9.1)	9 (7.7)
Complete blood count	81	12 (25.0)	13 (30.2)	2 (18.2)	43 (36.8)

Unit costs were derived from NHCHIS 2022 allowed amounts when available; otherwise public cash-price sources or manufacturer-listed prices were used, consistent with the original manuscript.

The most frequently captured conventional diagnostic tests were colonoscopy, upper endoscopy, and lactulose breath testing (**Table 4**). Colonoscopy was performed in 50.0% of IBS-Smart-only patients, 53.5% of Trio-Smart-only patients, 27.3% of patients who received both tests, and 60.7% of controls. Upper endoscopy was performed in 31.3%, 41.9%, 0.0%, and 48.7%, respectively. These absolute differences are clinically interpretable: compared with controls, the descriptive NNT to avert one colonoscopy was approximately 9.4 for IBS-Smart only, 13.9 for Trio-Smart only, and 3.0 for patients who received both tests. The corresponding NNT to avert one upper endoscopy was 5.7, 14.6, and 2.1, respectively (**Table 5**; **Supplementary Figure 3**).

**Table 5:** Direct descriptive comparison of endoscopy use and captured monthly cost versus control (new).

Tested cohort	Colonoscopy, %	Control, %	Absolute reduction, %	NNT to avert 1 colonoscopy	Upper endoscopy, %	Control, %	Absolute reduction, %	NNT to avert 1 EGD	Difference in total monthly captured cost, USD
IBS-Smart only	50.0	60.7	10.7	9.4	31.3	48.7	17.5	5.7	-593.54
Trio-Smart only	53.5	60.7	7.2	13.9	41.9	48.7	6.9	14.6	-577.52
Both tests	27.3	60.7	33.4	3.0	0.0	48.7	48.7	2.1	-598.56

NNT denotes the approximate number needed to test to avert one procedure based on absolute procedure-rate differences versus control. These values are descriptive only and should not be interpreted causally.

When follow-up time was normalized among patients first seen in 2018 or later, controls had the highest mean number of tests per patient per month (0.89) and the highest office visits per patient per month (1.22), compared with 0.59 and 0.35 in the IBS-Smart-only cohort, 0.43 and 0.41 in the Trio-Smart-only cohort, and 0.56 and 0.43 in the combined-testing cohort. Mean total diagnostic plus office-visit cost per patient per month was also highest in controls at $960.39, versus $366.85, $382.87, and $361.83 in the three tested cohorts. Relative to controls, the descriptive difference in total monthly captured cost was -$593.54 for IBS-Smart only, -$577.52 for Trio-Smart only, and -$598.56 for patients who received both tests (**Tables 5**, **6**).

**Table 6:** Utilization and captured cost per patient among patients first seen in 2018 or later (new).

Outcome	IBS-Smart only (n=37)	Trio-Smart only (n=31)	Both tests (n=10)	Control (n=70)
Tests per patient	5.22 (4.25)	4.00 (2.94)	3.30 (0.95)	3.63 (3.60)
Tests per patient per month	0.59 (0.63)	0.43 (0.55)	0.56 (0.70)	0.89 (1.55)
Test cost per patient per month, USD	260.97 (270.56)	259.71 (285.75)	232.56 (342.24)	591.40 (1313.07)
Office visits per patient	3.19 (3.92)	4.13 (2.53)	3.50 (1.96)	3.71 (2.76)
Office visits per patient per month	0.35 (0.54)	0.41 (0.65)	0.43 (0.50)	1.22 (2.47)
Office-visit cost per patient per month, USD	105.88 (164.08)	123.16 (196.90)	129.27 (152.33)	368.98 (749.40)
Total diagnostic + office cost per patient per month, USD	366.85 (370.67)	382.87 (346.07)	361.83 (475.64)	960.39 (1901.11)

Values are mean (SD). This table retains the original study restriction to patients whose first visit occurred in 2018 or later.

### Predictors of diagnostic costs

For analyses using propensity score-matched patients, 58 patients each were included in the IBS-Smart and control groups (based on the number of patients who had received an IBS-Smart test at the time the GLM analysis was performed). In independent t tests, patients in the IBS-Smart group were diagnosed with SIBO and had diarrhea significantly more often than patients in the control group (**Table 7**; **Supplementary Figures S4**, **S5**). SIBO, diarrhea, and migraine were the independent variables used in the GLM model to determine the effect that IBS-Smart testing has on the costs associated with diagnosing gastrointestinal disorders.

**Table 7:** Exploratory generalized linear model comparing IBS-Smart recipients with matched controls.

Predictor	Coefficient	Standard error	t value	P value
Intercept	7.90	0.11	68.88	<0.001
IBS-Smart test	−0.22	0.11	−1.98	0.050
SIBO	0.36	0.11	3.32	0.001
Diarrhea	0.19	0.11	1.72	0.088
Migraine	−0.21	0.12	−1.80	0.075

58 IBS-Smart recipients and 58 propensity-matched controls.

For the GLM, IBS-Smart testing was associated with lower total diagnostic cost (coefficient -0.22, P = 0.050), whereas SIBO was associated with higher cost (coefficient 0.36, P = 0.001). The GLM showed that IBS-Smart testing and SIBO were the most important factors predicting testing and office visit costs, with both being statistically significant (**Table 7**). The IBS-Smart test was associated with lower costs while SIBO was associated with higher costs. Additionally, the model predicts an incremental savings of $526 when a patient had the IBS-Smart test. In a separate GLM for trio-smart, the incremental savings for diagnostic test costs was $521.

### Chart level diagnostic persistence

Chart-level diagnostic persistence favored the tested cohorts. Stable diagnosis was observed in 79.2% of IBS-Smart-only patients and 90.7% of Trio-Smart-only patients, compared with 25.6% of controls. Among patients who received both tests, chart-level stability was 72.7% after IBS-Smart and 90.9% after Trio-Smart. Because this metric depends on charted diagnoses rather than on blinded adjudication, it is best interpreted as documentation stability rather than as confirmation of diagnostic correctness (**Table 8**).

**Table 8:** Chart-level diagnostic persistence.

Outcome	IBS-Smart only (n=48)	Trio-Smart only (n=43)	Both tests after IBS-Smart (n=11)	Both tests after Trio-Smart (n=11)	Control (n=117)
Stable diagnosis, n (%)	38 (79.2)	39 (90.7)	8 (72.7)	10 (90.9)	30 (25.6)
Changed diagnosis, n (%)	10 (20.8)	4 (9.3)	3 (27.3)	1 (9.1)	87 (74.4)

Diagnostic persistence reflects stability of charted diagnosis and should not be interpreted as the same construct as diagnostic accuracy.

### Medication use

Medication use decreased in several descriptive comparisons one year after testing. Among patients with IBS-Smart only, the mean number of all medications declined from 1.1 at test date to 0.6 at one year across all results combined. Among Trio-Smart-only patients, the mean number of all medications was 0.5 at the test date and 0.5 at one year across all results combined, with a small decline in positive-result patients from 0.4 to 0.3. Among patients who received both tests, mean all-medication counts declined from 1.2 to 0.6 after IBS-Smart and from 0.7 to 0.4 after Trio-Smart. Brand-medication counts also decreased in several subgroups (**Supplementary Table 1** and **2**). These findings remain descriptive because the dataset did not include indication-specific prescribing context for every change in therapy.

## Discussion

In this retrospective two-center real-world study, the use of IBS-Smart and/or Trio-Smart was associated with lower captured diagnostic-resource utilization, lower captured diagnostic plus gastroenterology office-visit costs, and greater chart-level diagnostic persistence compared with contemporaneous controls who did not receive either test. Controls underwent colonoscopy and upper endoscopy more frequently than the tested cohorts, and normalized monthly captured costs were also highest in controls. These findings suggest selective use of serologic and breath testing may influence diagnostic sequencing and reduce reliance on some downstream procedures. At the same time, the observational design and clinician-selected testing strategy require careful interpretation and should be understood as associations rather than proof of causal benefit.

It is important to note that these data do not change the current diagnostic framework for irritable bowel syndrome. IBS remains a disorder of gut-brain interaction and should be diagnosed using a positive clinical strategy grounded in Rome IV symptom criteria, careful history, assessment for alarm features, and limited targeted testing when clinically indicated. Current guidance supports a positive diagnosis rather than a diagnosis of exclusion, but that does not eliminate the need to consider specifically treatable diseases and disorders with an IBS phenotype, such as small intestinal bacterial overgrowth (SIBO), small intestinal fungal overgrowth (SIFO), inflammatory bowel disease, celiac disease, microscopic colitis, bile acid diarrhea, medication-related symptoms, selected infections, and other causes of chronic diarrhea or abdominal symptoms in the appropriate setting (1–4, 10). Accordingly, the present study should not be interpreted as evidence that proprietary testing can replace clinical reasoning, celiac serology, fecal calprotectin or C-reactive protein in selected patients with diarrhea symptoms, or other focused rule-out investigations.

This distinction is especially important when interpreting IBS-Smart. The anti-CdtB and anti-vinculin biomarkers were originally developed to help distinguish diarrhea-predominant IBS from inflammatory bowel disease in selected patients with chronic diarrhea, and the original validation work suggested relatively high specificity but only modest sensitivity (5). In other words, a positive result may increase confidence in a post-infectious IBS-D phenotype in the right clinical context, but a negative result does not exclude IBS, and the test does not obviate the need for appropriate rule-out testing when red flags or competing diagnoses are present. More recent external data have also shown that performance may be less robust in some populations than initially reported (6). For that reason, IBS-Smart is best viewed as an adjunctive tool that may support, rather than independently establish, a diagnosis in carefully selected patients.

A similarly cautious interpretation applies to Trio-Smart and breath testing more broadly. Hydrogen- and methane-based breath testing has been standardized through the North American consensus and remains the most practical noninvasive approach for evaluating suspected small intestinal bacterial overgrowth and methane-associated constipation in clinical practice (7, 8, 12). However, breath testing is also vulnerable to preanalytic and interpretive limitations (18), including patient preparation, substrate choice, oral hygiene, recent antibiotic use, intestinal transit, and the possibility of both false-positive and false-negative results (7, 8). Importantly, the AGA technical review on the evaluation of functional diarrhea and IBS-D did not include breath testing as part of the standard diarrhea work-up (4). Hydrogen sulfide measurement is a newer extension of breath testing, and although emerging data suggest symptom correlations, it should still be interpreted as phenotype-level information rather than a definitive end-diagnosis (7, 8). Thus, the present study should not be read as validating a broad “microbiome-first” diagnostic model for IBS.

One of the more interesting signals in this study was the greater chart-level diagnostic persistence in the tested cohorts than in controls. That observation is potentially relevant because durable diagnosis is one of the main practical goals of a positive IBS strategy. Recent follow-up data from secondary care suggest that a Rome IV IBS diagnosis made after limited, judicious investigation is generally safe and durable, with missed relevant organic gastrointestinal disease occurring infrequently (19). Even so, the outcome measured in the present study was persistence of the charted diagnosis, not adjudicated diagnostic accuracy. A stable chart label may reflect appropriate diagnostic confidence, but it may also reflect anchoring, clinician preference, or differences in coding behavior. For that reason, the diagnostic-persistence findings in this study are hypothesis-generating and supportive, not definitive proof that testing improved diagnostic correctness.

The economic findings should be interpreted with the same caution. The costing framework used here was intentionally pragmatic and limited to captured diagnostic tests and gastroenterology office visits from a payer-proxy perspective in 2022 US dollars (20). That approach is useful for illustrating how testing may alter near-term specialty-care utilization, but it does not represent the full economic burden of IBS care. Emergency department visits, primary care encounters, non-gastroenterology specialist care, productivity losses, dietary costs, travel time, and many downstream treatment expenditures were not captured. In addition, although the study included an exploratory propensity-matched generalized linear model, the authors do not overstate that model because of limited sensitivity analysis. The descriptive differences in procedure rates and normalized monthly costs are therefore more informative than any single modeled savings estimate.

This study nevertheless has several strengths. It reflects real-world practice across two centers, preserves separate IBS-Smart-only, Trio-Smart-only, combined-testing, and control cohorts, and addresses a clinically relevant question that is often discussed but infrequently studied using practice-based data. The analysis provides some interpretability by presenting direct one-to-one descriptive comparisons between each tested cohort and controls, clarifying the likely differential-diagnosis framework and more clinically aligned with how IBS is diagnosed and managed in contemporary gastroenterology practice.

From a practice perspective, the results support a stepwise rather than disruptive interpretation. In adults with symptoms suggestive of IBS and no alarm features, clinicians should still begin with a careful symptom history, Rome IV assessment, and focused testing guided by the predominant bowel pattern and clinical scenario (1–4, 10). Within that framework, IBS-Smart may be most relevant when post-infectious IBS-D is part of the differential, and Trio-Smart may be most relevant when bloating, gas, constipation, diarrhea, or suspected overgrowth phenotypes are likely to change management. The value proposition suggested by this study is therefore not that these tests replace standard evaluation, but that selective use in specialty care may help some clinicians avoid repeated visits or invasive procedures while maintaining diagnostic confidence. Future prospective studies should test that hypothesis more rigorously using standardized rule-out pathways, prespecified test-assignment criteria, full cost capture, and outcomes such as treatment response, rereferral, reinvestigation, and missed alternative diagnoses. Additionally, research should assess whether accurate and timely diagnosis of diseases and disorders presenting as the IBS phenotype leads to improved clinical outcomes and significant cost savings (21, 22).

### Limitations

There are several limitations with this study: retrospective design, unmeasured confounding, clinician-driven test selection, potential coding bias from ICD-based cohort identification, incomplete standardization of rule-out testing, small size of the combined-testing cohort, and lack of patient-centered outcomes such as symptom response, quality of life, or missed organic disease during follow-up.

## Conclusions

In this retrospective two-center study of adults with symptoms suggestive of irritable bowel syndrome, receipt of IBS-Smart and/or Trio-Smart was associated with lower captured diagnostic-resource utilization, lower captured diagnostic plus office-visit costs per patient per month, and greater chart-level diagnostic persistence than standard care without these tests. These findings should be interpreted cautiously. Because the study was observational, nonrandomized, and based on chart data, it cannot establish causality or show that proprietary serologic or breath testing replaces Rome IV-based clinical diagnosis, alarm-feature triage, or guideline-directed rule-out testing. The results are best understood as real-world signals that selective use of IBS-Smart and Trio-Smart may influence diagnostic sequencing and resource use in specialty practice, and that these hypotheses merit prospective validation.

## Data Availability

The datasets presented in this article are not publicly available because access is restricted by IRB and institutional requirements. Requests to access the datasets should be directed to Raf Magar, rmagar@ahrminc.com.
