# Supplementary material for: A real-world assessment of healthcare resource utilization following IBS-Smart ® and Trio-Smart ® testing in patients with suspected irritable bowel syndrome
Source: Front Gastroenterol (Lausanne). 2026 Jul 23;5:1681818. doi: 10.3389/fgstr.2026.1681818 (PMC13441705; doi:10.3389/fgstr.2026.1681818)
Supplement: Supplementary file 1 [file SupplementaryFile1.docx]

Supplementary Tables and Figures

Supplementary Table 1. All-medication counts at test date and one year after testing

| **Cohort/time anchor** | **Result category** | **n** | **Mean at test date** | **Mean 1 year after test** |
| --- | --- | --- | --- | --- |
| IBS-Smart only | Negative | 29 | 1.0 | 0.4 |
| IBS-Smart only | Positive | 11 | 1.4 | 0.7 |
| IBS-Smart only | Any result | 41 | 1.1 | 0.6 |
| Trio-Smart only | Negative | 15 | 0.5 | 0.5 |
| Trio-Smart only | Positive | 9 | 0.4 | 0.3 |
| Trio-Smart only | Any result | 24 | 0.5 | 0.5 |
| Both tests (after IBS-Smart) | Negative | 6 | 1.0 | 0.7 |
| Both tests (after IBS-Smart) | Positive | 3 | 1.7 | 0.3 |
| Both tests (after IBS-Smart) | Any result | 9 | 1.2 | 0.6 |
| Both tests (after Trio-Smart) | Negative | 5 | 0.8 | 0.8 |
| Both tests (after Trio-Smart) | Positive | 4 | 0.5 | 0.0 |
| Both tests (after Trio-Smart) | Any result | 9 | 0.7 | 0.4 |

Supplementary Table 2. Brand-medication counts at test date and one year after testing

| **Cohort/time anchor** | **Result category** | **n** | **Mean at test date** | **Mean 1 year after test** |
| --- | --- | --- | --- | --- |
| IBS-Smart only | Negative | 13 | 0.7 | N/A |
| IBS-Smart only | Positive | 6 | 0.9 | 0.2 |
| IBS-Smart only | Any result | 19 | 0.7 | 0.1 |
| Trio-Smart only | Negative | 13 | 0.1 | 0.2 |
| Trio-Smart only | Positive | 6 | 0.7 | 0.2 |
| Trio-Smart only | Any result | 19 | 0.3 | 0.2 |
| Both tests (after IBS-Smart) | Negative | 5 | 0.7 | 0.6 |
| Both tests (after IBS-Smart) | Positive | 4 | 0.7 | 0.0 |
| Both tests (after IBS-Smart) | Any result | 9 | 0.7 | 0.3 |
| Both tests (after Trio-Smart) | Negative | 5 | 0.0 | 0.6 |
| Both tests (after Trio-Smart) | Positive | 4 | 0.3 | 0.0 |
| Both tests (after Trio-Smart) | Any result | 9 | 0.1 | 0.3 |

Medication tables include only records with documented initiation and termination dates in the archived data

Supplementary Table 3. Independent-variable comparisons for the matched IBS-Smart analysis

| **Diagnosis/symptom** | **IBS-Smart (n=58)** | **Control (n=58)** | **P value** |
| --- | --- | --- | --- |
| SIBO | 42 (72.4) | 24 (41.4) | <0.001 |
| Diarrhea | 44 (75.9) | 34 (58.6) | <0.05 |
| Migraine | 12 (20.7) | 19 (32.8) | <0.15 |

Supplementary Table 4. Assay characteristics and guideline context relevant to interpretation

| **Test domain** | **What is known** | **Interpretive caution** |
| --- | --- | --- |
| IBS-Smart / anti-CdtB | Second-generation serologic marker used to help differentiate IBS-D from IBD rather than to diagnose all IBS phenotypes. In stabilized-epitope testing, anti-CdtB specificity was 93.5%, sensitivity 43.0%, and positive likelihood ratio 6.7 versus IBD. | Best interpreted in the appropriate pretest context after history, examination, and limited rule-out testing. Performance was reported against IBD rather than against all causes of chronic diarrhea. [10,11] |
| IBS-Smart / anti-vinculin | In the same second-generation study, anti-vinculin specificity was 90.9%, sensitivity 52.2%, and positive likelihood ratio 5.7 versus IBD. When both antibodies were positive and a 57% pretest probability was assumed, post-test probability exceeded 98%. | Useful as an adjunct, not a stand-alone substitute for clinical diagnosis. Performance may differ in lower-prevalence settings. [11] |
| Hydrogen or lactulose/glucose breath testing | North American consensus and ACG SIBO guideline support standardized hydrogen/methane breath testing as a noninvasive option for selected patients with bloating, IBS-like symptoms, or constipation. A rise in hydrogen ≥20 ppm by 90 minutes is commonly used for SIBO; methane ≥10 ppm at any time is considered methane-positive. | Evidence quality is low/very low, and diagnostic accuracy varies by substrate, cut point, and reference standard. Breath testing supports hypothesis-driven evaluation of SIBO/IMO rather than routine universal IBS work-up. [12,13] |
| Hydrogen sulfide (H2S) breath testing / ISO | Three-gas breath testing can characterize H2S-associated breath patterns, but there is no universally accepted pooled sensitivity/specificity estimate and H2S testing is still emerging relative to hydrogen/methane standards. | In this manuscript, ISO refers to intestinal sulfide overproduction reported on breath testing, not intestinal spirochetosis. This remains an investigational or evolving phenotype rather than an established end-diagnosis. [14,15] |
| Pre-analytic considerations | Breath testing requires careful preparation, including diet restriction, overnight fasting, and avoidance of recent antibiotics; laxatives, promotility agents, oral hygiene, and intestinal transit can affect results. | Nonadherence can generate false-positive or uninterpretable results, which is one reason current guidelines emphasize standardization rather than indiscriminate use. [12,16,17] |

**Supplementary Figure 1.** Study design

d

Variable follow-up period

a

Index date (visit 1)

2018

2019

2020

2021

2022

2023

b

Post-index visits

c

Last visit

IBS-Smart commercially available

Oct 2018

Trio-Smart commercially available

Oct 2020

Q4 2022

Data collection period

Q3 2023

e

Study period

aIndex is the patient’s first office visit, recorded by month and year of visit; bPost-

index visits were recorded according to days from index date; patients had a minimum of 3 visits (including the index and last visits); cLast visit during the study period denotes end of the variable follow-up period; dFor each patient, medical records were reviewed from their first to last visit within the study period; eStudy period was defined by the year of the earliest patient visit to the last patient visit reviewed in the study; specific dates were not recorded to maintain patient confidentiality.

Supplementary Figure 3. Covariance Balance Plot for IBS-Smart

Figure above presents a love plot for the **IBS-Smart group** summarizing covariate balance before (unadjusted; red) and after (adjusted; teal) the propensity-score–based adjustment for the IBS-Smart group and matched controls.

The x‑axis shows the (standardized) mean difference between comparison groups for each covariate, with the vertical line at 0 indicating perfect balance.

In the unadjusted sample, several baseline characteristics exhibited meaningful imbalance, with standardized mean differences generally in the range of approximately −0.1 to −0.35 for variables such as age, history_before_2019, MCAS_first_visit, IBS_D_first_visit, and refer_y_. In addition, the propensity score distance measure showed substantial separation between groups (large positive mean difference), consistent with strong baseline differences in treatment assignment prior to adjustment.

After adjustment, the covariate means differences moved markedly toward zero across all measured characteristics, indicating improved balance between groups. Most covariates cluster tightly around 0, suggesting the adjustment procedure was effective in reducing systematic baseline differences; the largest residual differences appear modest relative to the unadjusted sample.

Supplementary Figure 4. Covariance Balance Plot for Trio-Smart

Figure displays a covariate balance plot (love plot) for the TRIO-Smart group comparing standardized mean differences between study groups before (unadjusted; red) and after (adjusted; teal) application of the propensity-score–based adjustment. The variables assessed include history_before_2019, ibs_first_visit, SIBO_first_visit, and the propensity score distance metric.
